# Yeast hEST1A/B (SMG5/6)–Like Proteins Contribute to Environment-Sensing Adaptive Gene Expression Responses

**DOI:** 10.1534/g3.113.006924

**Published:** 2013-10-01

**Authors:** Xianning Lai, Traude Beilharz, Wei-Chun Au, Andrew Hammet, Thomas Preiss, Munira A. Basrai, Jörg Heierhorst

**Affiliations:** * St. Vincent’s Institute of Medical Research, Melbourne, Victoria 3065, Australia; † Department of Medicine, St. Vincent’s Hospital, The University of Melbourne, Melbourne, Victoria 3065, Australia; ‡ Victor Chang Cardiac Research Institute, Sydney, New South Wales 2010, Australia; § Center for Cancer Research, National Cancer Institute, National Institutes of Health, Bethesda, Maryland 20889

**Keywords:** hEST1, SMG5, SMG6, gene expression, genetic interactions

## Abstract

During its natural life cycle, budding yeast (*Saccharomyces cerevisiae*) has to adapt to drastically changing environments, but how environmental-sensing pathways are linked to adaptive gene expression changes remains incompletely understood. Here, we describe two closely related yeast hEST1A-B (SMG5-6)–like proteins termed Esl1 and Esl2 that contain a 14-3-3–like domain and a putative PilT N-terminus ribonuclease domain. We found that, unlike their metazoan orthologs, Esl1 and Esl2 were not involved in nonsense-mediated mRNA decay or telomere maintenance pathways. However, in genome-wide expression array analyses, absence of Esl1 and Esl2 led to more than two-fold deregulation of ∼50 transcripts, most of which were expressed inversely to the appropriate metabolic response to environmental nutrient supply; for instance, normally glucose-repressed genes were derepressed in *esl1Δ esl2Δ* double mutants during growth in a high-glucose environment. Likewise, in a genome-wide synthetic gene array screen, *esl1Δ esl2Δ* double mutants were synthetic sick with null mutations for Rim8 and Dfg16, which form the environmental-sensing complex of the Rim101 pH response gene expression pathway. Overall, these results suggest that Esl1 and Esl2 contribute to the regulation of adaptive gene expression responses of environmental sensing pathways.

hEST1A/B (SMG5/6) are structurally closely related bifunctional metazoan proteins with roles in telomere maintenance and in the nonsense-mediated mRNA decay (NMD) pathway that degrades mRNAs containing premature stop codons during quality-control pioneer rounds of translation in the nucleus. hEST1A/B (SMG5/6) contain a central 14-3-3–like domain (Fukuhara *et al.* 2005) that may mediate protein–protein interactions for regulation of the key NMD factor UPF1 (Anders *et al.* 2003; Chiu *et al.* 2003; Ohnishi *et al.* 2003) and a C-terminal PilT N-terminus (PIN) domain that provides endoribonuclease activity toward degradation of NMD substrates (Eberle *et al.* 2009; Huntzinger *et al.* 2008). Another related protein, hEST1C (SMG7), also contains a central 14-3-3 domain but lacks the C-terminal PIN domain. NMD proteins such as hEST1A/B (SMG5/6) are highly enriched at telomeres (Reichenbach *et al.* 2003; Snow *et al.* 2003) and negatively regulate the expression of telomeric repeat–containing RNA (Schoeftner and Blasco 2008), which may explain the crosstalk between NMD and telomere maintenance pathways.

The name hEST1A-C relates to the similarity of these proteins to the yeast telomerase subunit Est1 within the 14-3-3–like domain, which therefore is also referred to as the Est-one-homology domain (Beernink *et al.* 2003; Chiu *et al.* 2003; Reichenbach *et al.* 2003; Snow *et al.* 2003). However, it recently has been shown that the yeast NMD factor Ebs1 is the structural and functional ortholog of hEST1C (SMG7) (Luke *et al.* 2007), and no yeast counterparts for hEST1A/B (SMG5/6) previously have been identified. During database mining attempts to identify potential cofactors of a yeast protein with DNA damage and telomere-related functions, Mdt1/Pin4 (Pike and Heierhorst 2007; Pike *et al.* 2004; Traven *et al.* 2010), we noticed that a large-scale two-hybrid screen (Uetz *et al.* 2000) had found it to interact with two uncharacterized Est-one-homology and PIN domain–containing open reading frames Yil151c and Ykr096w. Here, we show that Yil151c and Ykr096w are structural orthologs of hEST1A-B/SMG5-6 and have thus named them Esl1 and Esl2 (ESL = EST/SMG–like). Surprisingly, we found that Esl1 and Esl2 have no apparent telomere-related or NMD functions but instead are involved in the expression of a small subset of genes, including hexose and amino acid metabolism–related genes, during adaptation to nutrient supply by the environment.

## Materials and Methods

### Yeast strains

All yeast strains used in this study are listed in Table 1 and were derived from W303-1A, unless otherwise indicated. Gene disruptions and C-terminal tagging were performed using a technique mediated by polymerase chain reaction (PCR) (Longtine *et al.* 1998). Nuclease-dead mutants were generated by PCR-based site-directed mutagenesis as described (Erdeniz *et al.* 1997; Pike *et al.* 2003). Synthetic lethality screening and tetrad dissection were performed in the BY4741 background. Subtelomeric gene silencing assays were performed using the UCC3505 strain (Singer and Gottschling 1994). Experiments were performed in YPD medium (1% yeast extract, 2% peptone, 2% glucose) at 30°, except for selection against *petites*, for which cells were plated on YPG (1% yeast extract, 2% peptone, 3% glycerol).

**Table 1:** Yeast strains used in this study

Strain	Genotype	Reference
Y52 (W303-1a)	MATa *ade2-1 can1-100 leu2-3*, *122 trp1-1 ura3-1 RAD5*	Zhao *et al.* 1998
Y829	Y52 *esl1*Δ::*KAN*	This study
Y830	Y52 *esl2*Δ::*NAT*	This study
Y831	Y52 *esl1*Δ::*KAN esl2*Δ::*NAT*	This study
Y1113	Y52 *upf1*Δ::*URA3*	This study
Y1115	Y52 *esl1*Δ::*KAN upf1*Δ::*URA3*	This study
Y1117	Y52 *esl2*Δ::*NAT upf1*Δ::*URA3*	This study
Y1119	Y52 *esl1*Δ::*KAN esl2*Δ::*NAT upf1*Δ::*URA3*	This study
Y1280	Y52 *esl1-nd*	This study
Y1282	Y52 *esl2-nd*	This study
Y1284	Y52 *esl2-nd*	This study
Y1333	Y52 *trf4*Δ::*NAT*	This study
Y1335	Y52 *rim8*Δ::*NAT*	This study
Y1342	Y52 *esl1-nd esl2-nd*	This study
Y1344	Y52 *esl1-nd esl2-nd*	This study
Y996 (Y7092)	MATα *his3Δ1 leu2Δ0 ura3Δ0 met15Δ0 lyp1Δ cyh2 can1*Δ::*STE2pr-SpHIS5*	Tong and Boone 2006
Y1099	Y996 *esl1*Δ::*NAT esl2*Δ::*URA3*	This study
Y32 (BY4741)	MATa *his3*Δ*1 leu2*Δ*0 met15*Δ*0 ura3*Δ*0*	Brachmann *et al.* 1998
Y1289	Y32 *esl1*Δ::*NAT*	This study
Y1290	Y32 *esl2*Δ::*URA3*	This study
Y1291	Y32 *esl1*Δ::*NAT esl2*Δ::*URA3*	This study
Y1407	Y32 *esl1-nd*	This study
Y1408	Y32 *esl1-nd*	This study
Y1410	Y32 *esl1-nd*	This study
Y1417	Y32 *esl1-nd esl2-nd*	This study
Y1418	Y32 *esl1-nd esl2-nd*	This study
Y219 (JKM179)	*ade1 leu2-3,112 lys5 trp1*::*hisG ura3-52 hml*Δ::*ADE1 hmr*Δ::*ADE1 ade3*::*GAL-HO*	Lee *et al.* 1998
Y496 (TGI354)	*ade1 leu2-3,112 lys5 trp1*::*hisG ura3-52 hml*Δ::*ADE1 hmr*Δ::*ADE1 ade3*::*GAL-HO MATa-inc arg5,6*::*MATa-HPH*	Ira *et al.* 2003

### Solid medium plate assays

Overnight cultures were diluted to a starting density of A_600_ = 0.5 and were spotted in 10-fold serial dilutions onto YPD plates or medium containing various concentrations of drugs as indicated. Plates were incubated for 3–5 days at 30°.

### Nucleic acids blots

Cellular DNA and RNA were prepared by phenol–chloroform extraction. RNA was separated by electrophoresis at 80 V in 1.2% (w/v) agarose gels containing 1× MOPS buffer and 6.3% formaldehyde with buffer recirculation. Agarose gels for DNA analysis contained 0.5× TAE. Nucleic acids were transferred overnight by capillary transfer to nylon membranes using 10× SSC buffer. Membranes were incubated with radioactively labeled probes, exposed to phosphorimager screens, and analyzed using Molecular Dynamics ImageQuant software. For analysis of telomere lengths, genomic DNA was subjected to *Xho*I restriction endonuclease digestion at 37° for 4 hr as described (Pike and Heierhorst 2007; Traven *et al.* 2010).

### Synthetic genetic array analysis

The screen for synthetic sick/lethal interactions was performed for the query strain *esl1Δ esl2*Δ (Y1099) according to the method described (Tong and Boone 2006). Positive interactions from the screen were individually validated by tetrad dissections on YPD plates.

### Senescence assays

Sporulation cultures were digested with Zymolyase 20T in sorbitol buffer and tetrads were dissected on YPD plates using a dissection microscope; 10^6^ cells of freshly dissected spores were allowed to grow for 24 hr in YPD media at 30°. In exactly 24-hr intervals, cell densities were determined by hemocytometer counts of sonicated aliquots before redilution to 10^5^ cells/ml. Approximately 200–400 cells were plated on YPD each day and colonies were counted after 3 to 4 days.

### Multiple sequence alignment

The Basic Local Alignment Search Tool on the National Center for Biotechnology Information web site (http://blast.ncbi.nlm.nih.gov/Blast.cgi) was used to identify regions of similarity between biological sequences. Multiple sequence alignments were generated with ClustalW on the European Bioinformatics Institute web site (http://www.ebi.ac.uk/Tools/msa/clustalw2/). All conserved and similar residues in the multiple sequence alignments were shaded using BoxShade 3.2 on the Swiss EMBnet server (http://www.ch.embnet.org/software/BOX_form.html).

### DNA microarray

Total RNA was prepared from YPD log-phase cultures of wild-type and *esl1Δ esl2Δ* double mutants in the W303-1A background. cDNA synthesis and two-color hybridization on yeast 8×15K format slides were performed by the Ramaciotti Centre for Gene Function Analysis (University of New South Wales). Data analysis was performed using the GeneSpring software (Agilent). Gene ontology enrichment analysis was performed using FuncAssociate 2.0 software (Berriz *et al.* 2009). The array data have been deposited in the National Center for Biotechnology Information Gene Expression Omnibus (GEO accession number GSE48956).

### Reverse-transcription PCR

Reverse-transcription PCR was performed using the method described previously (Beilharz and Preiss 2009).

## Results

### Identification of Esl1 and Esl2 as yeast orthologs of hEST1A-B/SMG5-6

**Figure 1:**
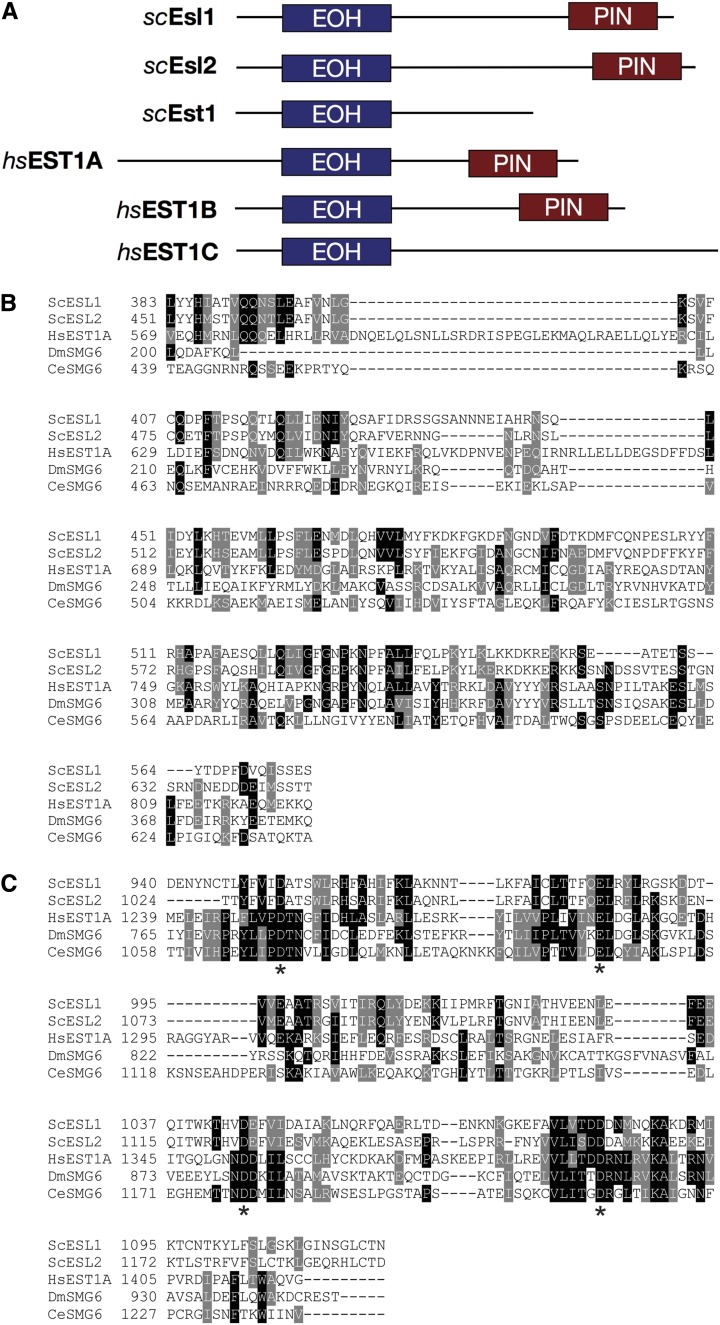
Comparison of Esl1 and Esl2 with metazon hEST1A/B (SMG6/5) proteins. (A) Schematic illustration of domain topology of Esl1, Esl2, Est1, and hEST1A-C. (B) Multiple sequence alignment of the Est-one-homology (EOH) domains of *S. cerevisiae* Esl1, Esl2, Est1, hEST1A, *Drosophila melanogaster* SMG6, and *C. elegans* SMG6. (C) Multiple sequence alignment of the PIN nuclease domains of *S. cerevisiae* Esl1, Esl2, Est1, hEST1A, *D. melanogaster* SMG6, and *C. elegans* SMG6. *The four conserved acidic residues required for nuclease function. For a similar alignment of Esl1 and Esl2 with the other budding yeast PilT N-terminus (PIN) domain–containing proteins, see Rüther *et al.* 2006.

During database searches we noted that two of the reported Mdt1-interacting proteins (Uetz *et al.* 2000), the previously uncharacterized yeast open reading frames Yil151c and Ykr096w, share >70% similarity with each other along their entire polypeptide sequence. Interestingly, during Basic Local Alignment Search Tool searches for metazoan orthologs, we noticed that these two proteins also share extensive similarity (∼45% overall) with human hEST1A/B and *Drosophila* and *Caenohabditis elegans* SMG5/6 proteins. Importantly, this similarity encompassed the region corresponding to the 14-3-3–like Est-one-homology domain (45–51% similarity; Figure 1, A and B) and the C-terminal PIN endonuclease domain (49–53% similarity; Figure 1, A and C) with complete conservation of four critical D/E residues required for nuclease activity of the PIN-domain proteins (asterisks in Figure 1C). Based on the structural similarities to hEST1A-B/SMG5-6, we have named Yil151c and Ykr096w Esl1 and Esl2 (ESL = EST/SMG–like), respectively.

### Esl1 and Esl2 do not have telomere-related or NMD-related functions

**Figure 2:**
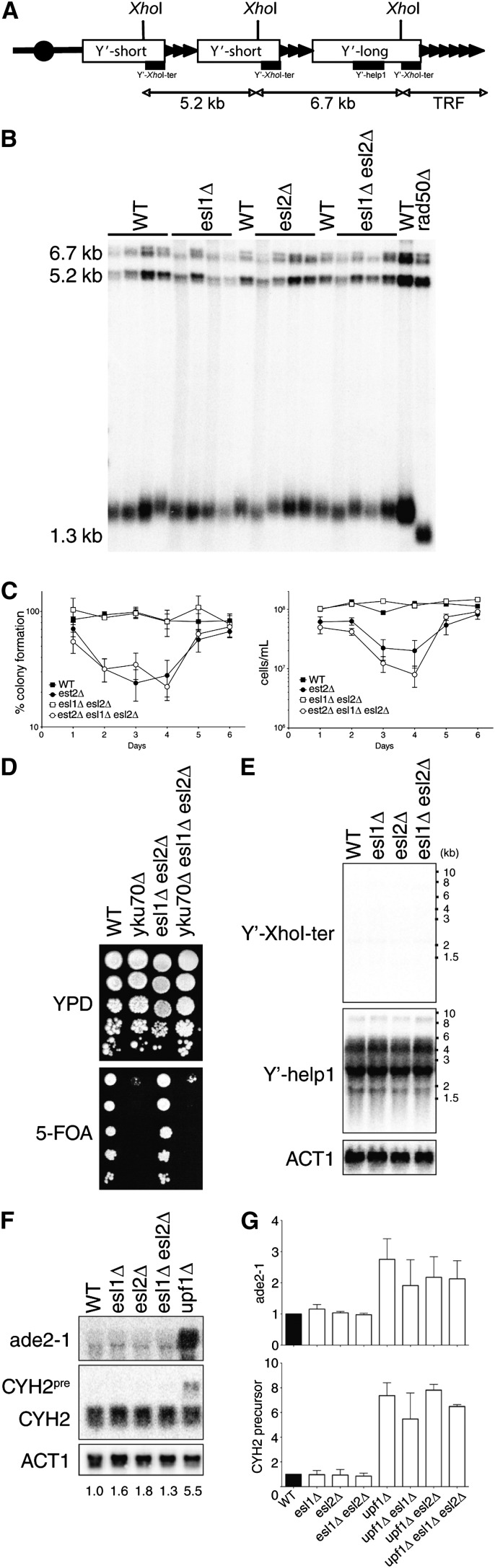
Esl1 and Esl2 do not have telomere-related or nonsense-mediated mRNA decay (NMD)-related functions. (A) Schematic illustration of assays to measure telomere length and gene expression. *Xho*I restriction sites and Y′-*Xho*I-ter probe used for Southern analysis are indicated. The probe detects the 5.2-kb Y′-short element, 6.7-kb Y′-long element, and the terminal restriction fragment (TRF). The Y′-help1 probe used for northern analysis in (E) is also indicated. (B) Southern blot analysis of independent clones of wild-type (WT), *esl1Δ*, *esl2Δ*, and *esl1Δ esl2Δ*. (C) Cultures were inoculated with ∼10^5^ cells/ml and back-diluted to 10^5^ cells/ml in exactly 24-hr intervals (right). Approximately 200–400 cells from daily cultures in the left panel were plated on YPD. Plates were incubated for 3 days at 30°C and percentage of plated cells able to form a colony was determined (left). Results are means ± SE from three independent wild-type (WT) and *esl1Δ esl2Δ* clones and seven independent *est2Δ* and *esl1Δ esl2Δ est2Δ* clones. (D) Ten-fold serial dilutions of WT, *yku70Δ*, *esl1Δ esl2Δ*, and *yku70Δ esl1Δ esl2Δ* were spotted onto YPD and 5′-fluororotic acid (5-FOA) plates. Plates were incubated 3–4 days at 30°C. (E) Northern blot analysis of telomeric repeat–containing RNAs (TERRAs; measured by the Y′-*Xho*I-ter probe) and Y′ element encoded helicase (measured by the *Y′-help1* probe). *ACT1* is used as the loading control. Note that no TERRA signal was detectable in any of the strains under basal conditions. (F) Northern analysis to measure NMD substrate levels in WT, *esl1Δ*, *esl2Δ*, *esl1Δ esl2Δ*, and *upf1Δ*. (G) Quantification of northern analysis in (F) normalized to actin levels. Results are means ± SE from three independent experiments.

To test if the structural similarities extend to similar protein functions, we monitored *esl1Δ* and *esl2Δ* single-null and *esl1Δ esl2Δ* double-null mutants for telomere-related and NMD-related defects (Pike and Heierhorst 2007; Traven *et al.* 2010). Telomere length in several independent *esl1Δ*, *esl2Δ* and *esl1Δ esl2Δ* clones were within the range of the wild-type, in contrast to *rad50Δ* mutants, which were included as a control for very short but stable telomeres (Figure 2, A and B), indicating that *ESL1* and *ESL2* do not contribute to normal telomerase-dependent telomere length control. In the absence of telomerase, cells progressively senesce until a small subpopulation of so-called postsenescence survivors emerges that has switched to recombination-dependent alternative lengthening of telomeres pathways (Lundblad and Blackburn 1993). To determine if Esl1 and Esl2 are involved in alternative lengthening of telomeres, we deleted the gene for the catalytic subunit of telomerase, *EST2*, for senescence assays. However, the kinetics of the onset of senescence and the emergence of postsenescence survivors with normal proliferative capacity and colony formation were similar for *esl1Δ esl2Δ est2Δ* triple mutants compared with *est2Δ* alone (Figure 2C), and both cases of postsenescent colonies predominantly comprised the more efficient type II survivors subtype (data not shown). Taken together, these results indicate that Esl1 and Esl2 are not required for telomerase-dependent or alternative telomere maintenance mechanisms.

Apart from telomere length regulation, telomere-associated proteins may be involved in maintaining the heterochromatin structure of telomeres and transcriptional repression of telomere-proximal genes (Baur *et al.* 2001; Blasco 2007; Gottschling *et al.* 1990). To assess if Esl1 and Esl2 affect telomere structure, we monitored 5′-fluororotic acid (5-FOA) sensitivity of strains containing a subtelomeric *URA3* reporter gene. Ura3 converts 5-FOA to a toxic metabolite and, consequently, *yku70Δ* control cells that are unable to silence the subtelomeric *URA3* reporter (Gottschling *et al.* 1990) were unable to grow on 5-FOA plates (Figure 2D). In contrast, *esl1Δ esl2Δ* mutants were able to grow on 5-FOA similar to the wild-type, and deletion of *ESL1* and *ESL2* did not affect the 5-FOA sensitivity (Figure 2D). In addition, there was no accumulation of natural subtelomere or telomere-derived transcripts, such as Y′-help and telomeric repeat–containing RNAs, in *esl1Δ*, *esl2Δ* and *esl1Δ esl2Δ* mutants (Figure 2E). Thus, Esl1 and Esl2 appear to be dispensable for maintenance of telomere structure.

To determine if Esl1 and Esl2 have NMD functions, we first measured expression levels of the endogenous nonsense-mutated *ade2-1* locus. In contrast to the *bona fide* NMD-deficient *upf1Δ* control (He *et al.* 1997), there was no accumulation of *ade2-1* transcripts in *esl1Δ* and/or *esl2Δ* mutants (Figure 2F). The NMD pathway also degrades unspliced transcripts and, similar to the *ade2-1* result, there was no accumulation of the unspliced pre-*CYH2* mRNA in *esl1Δ* and/or *esl2Δ* mutants (Figure 2G). Thus, based on these two independent assays, *ESL1* and *ESL2* do not seem to have NMD-related functions.

### *ESL1* and *ESL2* contribute to some genome stability functions in a nuclease domain–dependent manner

**Figure 3:**
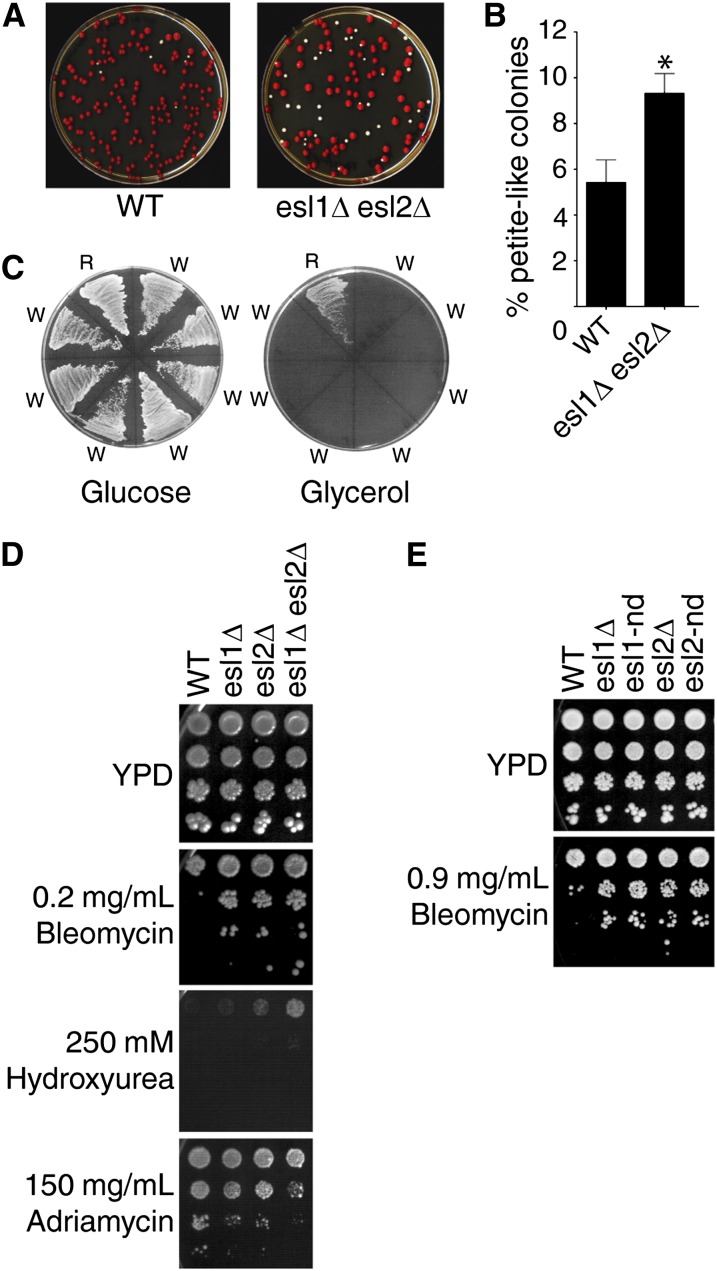
Genome stability functions of Esl1 and Esl2. (A) Freshly sporulated cultures were plated on YPD and plates were incubated for 3–4 days at 30°C. (B) Quantification of the percentage of white colonies formed on YPD plates for the indicated genotypes. Data are means ± SE. *p < 0.005, n = 21, two-tailed Student *t* test. (C) Seven randomly picked white *petite*-like colonies (W) and a single red colony (R) were restreaked on glucose (left) and glycerol (right) plates. (D and E) Ten-fold serial dilutions of the indicated strains were spotted onto YPD plates and YPD plates containing the indicated concentrations of various DNA-damaging drugs. The *esl1-nd* mutant carries two amino acid substitutions (D952N and E982Q) while the *esl2-nd* mutant carries a single amino acid substitution (D1123N). Plates were incubated for 3–4 days at 30°C. WT, wild-type.

Cells containing the *ade2-1* nonsense mutation have a pink color (Figure 3A, WT). During routine propagation of *esl1Δ esl2*Δ double mutants, we noticed that culture plates had an increased incidence of white colonies, which is often attributable to the spontaneous accumulation of mitochondrial DNA mutations (Zhao *et al.* 1998) (Figure 3, A and B). All white *esl1Δ esl2*Δ colonies failed to grow on YP-glycerol plates (Figure 3C) on which respiration-deficient mitochondrial *petite* mutants are unviable, indicating that *ESL1* and *ESL2* contribute to maintenance of mitochondrial genome stability.

To test if Esl1 and Esl2 may be involved in additional genome stability functions, we monitored their sensitivity to a range of genotoxic agents. In drop tests on plates containing DNA-damaging agents, *esl1Δ* and/or *esl2Δ* mutants grew ∼100-fold better in the presence of 0.2 µg/ml bleomycin, ∼10-fold better on 250 mM hydroxyurea, and ∼10-fold worse on 150 µg/ml adriamycin compared with the wild-type (Figure 3D). In all cases, the effect was more pronounced when *ESL1* and *ESL2* were simultaneously deleted, suggesting functional redundancy between the two proteins. To determine if these phenotypes were attributable to a potential PIN nuclease function, similar drop tests were performed using “nuclease-dead” *esl1* and e*sl2* mutants containing amino acid substitutions of at least one of the four critical conserved residues whose mutation previously has been shown to abrogate the nuclease activity of other PIN domain proteins (Dziembowski *et al.* 2007; Schneider *et al.* 2007; Skruzny *et al.* 2009). In these assays, the nuclease-dead mutants phenocopied the bleomycin sensitivity of the *esl1*Δ or *esl2*Δ mutants (Figure 3E). Altogether, the results indicate that Esl1 and Esl2 contribute to the maintenance of genome stability in a manner that, at least in some cases, depends on their nuclease function.

### Loss of *ESL1* and of *ESL2* lead to impaired genetic fitness with *trf4Δ*, *rim8Δ*, and *dfg16Δ*

**Figure 4:**
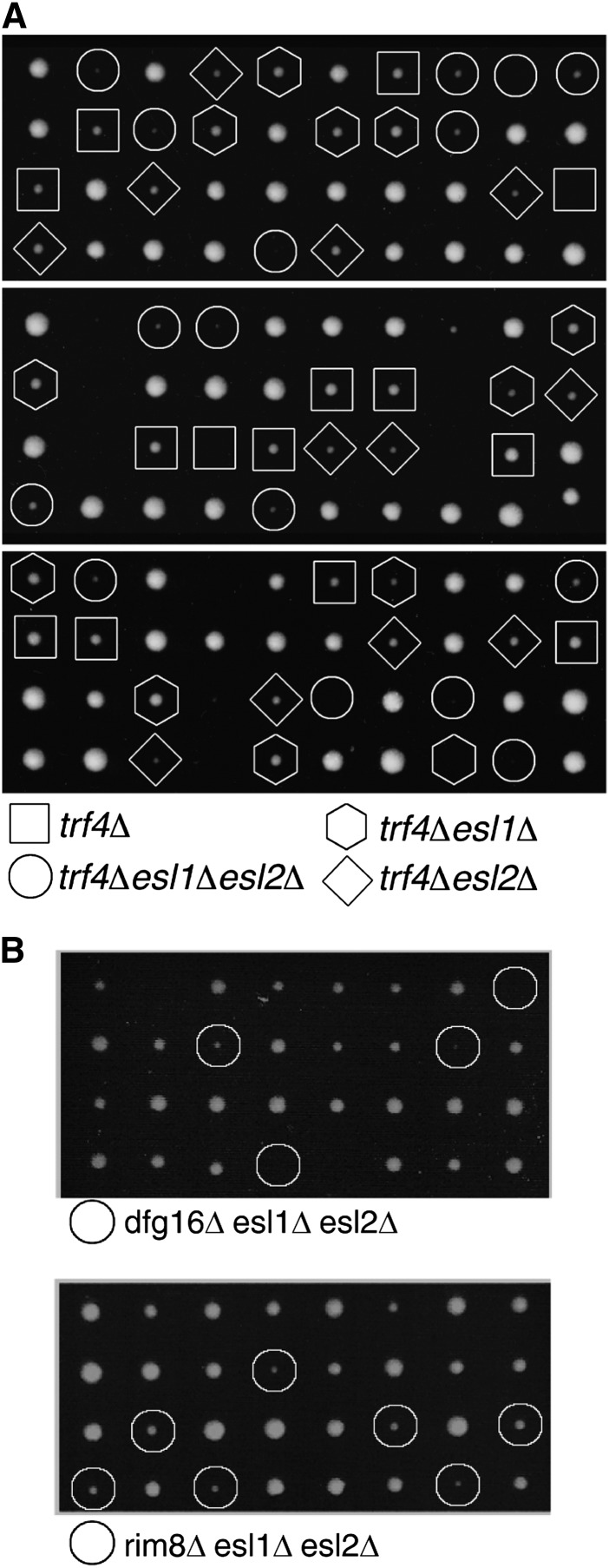
Genetic interactions of *ESL1* and *ESL*2 with *TRF4* and *DFG16/RIM8*. Tetrad dissection of compound heterozygous diploid strains for *esl1Δ* and *esl2Δ* as well as *trf4*Δ (A) or with *rim8*Δ or *dfg16*Δ (B). Genotypes of spores are indicated in (A); circles in (B) denote triple mutants. Plates were incubated for 3–4 days at 30°C.

As an unbiased approach to identify possible cellular functions of Esl1 and Esl2, a synthetic gene array screen was performed. For this purpose, an *esl1Δ esl2Δ* double-mutant strain was mated with the complete set of haploid-viable deletion yeast deletion mutants (Tong and Boone 2006), sporulated, and then plated on three different types of selective media to detect synthetic genetic interactions of *esl1*Δ or *esl2*Δ single mutants and *esl1Δ esl2*Δ double mutants. In the high-throughput screening format, *esl1*Δ was synthetic sick with four other deletions, *esl2*Δ was sick or lethal with 12 other deletions, and *esl1Δ esl2*Δ double mutants were synthetic sick or lethal with another seven gene deletions (Table 2). The genetic interactions identified by this approach are enriched in the functional categories phosphatidylinositol-3 phosphate binding (GO:0032266; adjusted p = 0.004) and endosome (GO:0005768; adjusted p = 0.008). Surprisingly, only three of the interactions, with *trf4Δ*, *rim8Δ*, and *dfg16Δ*, also were observed by manual tetrad dissection analysis on rich YPD medium (Figure 4), presumably because the less restrictive growth conditions compared with synthetic medium (plus antibiotics) select against somewhat weaker genetic interactions. Moreover, in all three cases, the synthetic growth defect on dissection plates was stronger with *esl1Δ esl2Δ* double mutants compared with single mutants (Figure 4), indicative of functional redundancy between Esl1 and Esl2. Interestingly, two of these *ESL1* and *ESL2* interactors, Dfg16 and Rim8 (Figure 4B), also physically interact as the G-protein-coupled receptor and β-arrestin–like adaptor in the Rim101 pathway that regulates the expression of pH-responsive genes in the adaptive response to alkaline environments (Lamb and Mitchell 2003; Lamb *et al.* 2001; Lin *et al.* 2008). However, Trf4 (Figure 4A) is a noncanonical poly(A) polymerase that forms part of the TRAMP complex involved in exosome-dependent RNA degradation (LaCava *et al.* 2005; Vanacova *et al.* 2005; Wyers *et al.* 2005), which is interesting in view of the notion that all other PIN domain-containing proteins characterized to date exert ribonuclease activity *in vitro* and/or *in vivo* (Bleichert *et al.* 2006; Eberle *et al.* 2009; Fatica *et al.* 2004; Huntzinger *et al.* 2008; Schaeffer *et al.* 2009).

**Table 2:** Synthetic genetic interactions of esl1 and esl2 in a synthetic gene array screen

ORF	Gene	*esl1Δ*	*esl2Δ*	*esl1Δ esl2Δ*
YBL016W	*FUS3*	Sick	ND	ND
YIR023W	*DAL81*	Sick	ND	ND
YJL036W	*SNX4*	Sick	ND	ND
YOL115W	*TRF4*	Lethal	ND	ND
YBR026C	*ETR1*	ND	Sick	Sick
YBR131W	*CCZ1*	ND	Sick	Sick
YCR063W	*BUD31*	ND	Sick	Sick
YDR074W	*TPS2*	ND	Sick	Sick
YGL212W	*VAM7*	ND	Sick	Sick
YJL204C	*RCY1*	ND	Sick	Sick
YOR106W	*VAM3*	ND	Sick	Sick
YOR030W	*DFG16*	ND	Sick	Sick
YOR132W	*VPS17*	ND	Sick	Sick
YOL012C	*HTZ1*	ND	Sick	Sick
YDR080W	*VPS41*	ND	Sick	Lethal
YER071C	*YER071C*	ND	Sick	Lethal
YFL010C	*WWM1*	ND	Normal	Sick
YGL050W	*TYW3*	ND	Normal	Sick
YGL045W	*RIM8*	ND	Normal	Sick
YGR164W	*YGR164W*	ND	Normal	Sick
YFL003C	*MSH4*	ND	Normal	Lethal
YOL116W	*MSN1*	ND	Normal	Lethal
YDR293C	*SSD1*	ND	Normal	Lethal

Sick/lethal interactions with single or double mutants were screened and scored visually. ND, not determined.

### Deregulation of hexose and one-carbon metabolism genes in *esl1Δ esl2Δ* mutants

**Figure 5:**
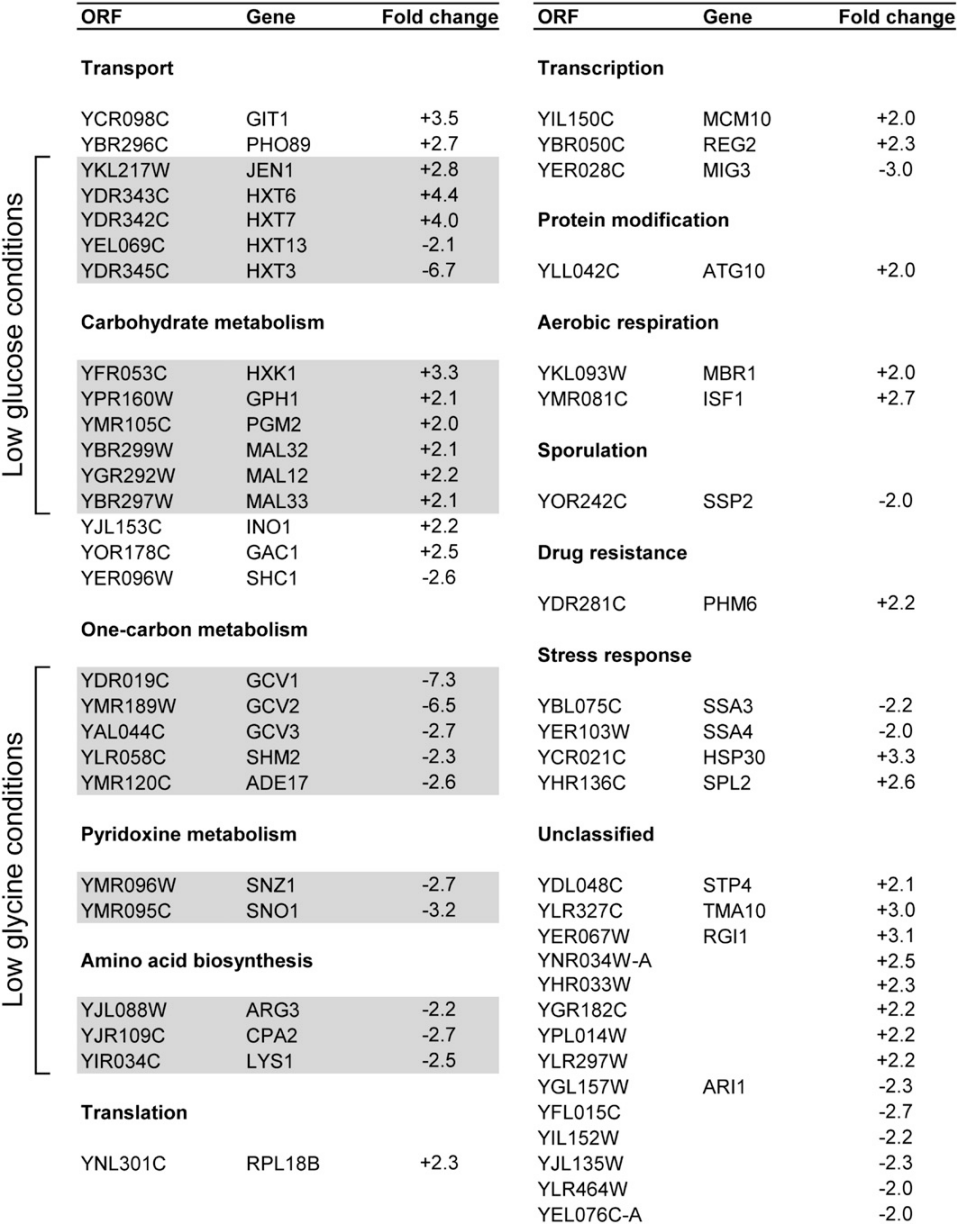
Classification of deregulated transcripts from the microarray. Gray boxes highlight genes that were expressed at levels that were opposite of what was expected for the glucose and glycine concentrations used.

**Figure 6:**
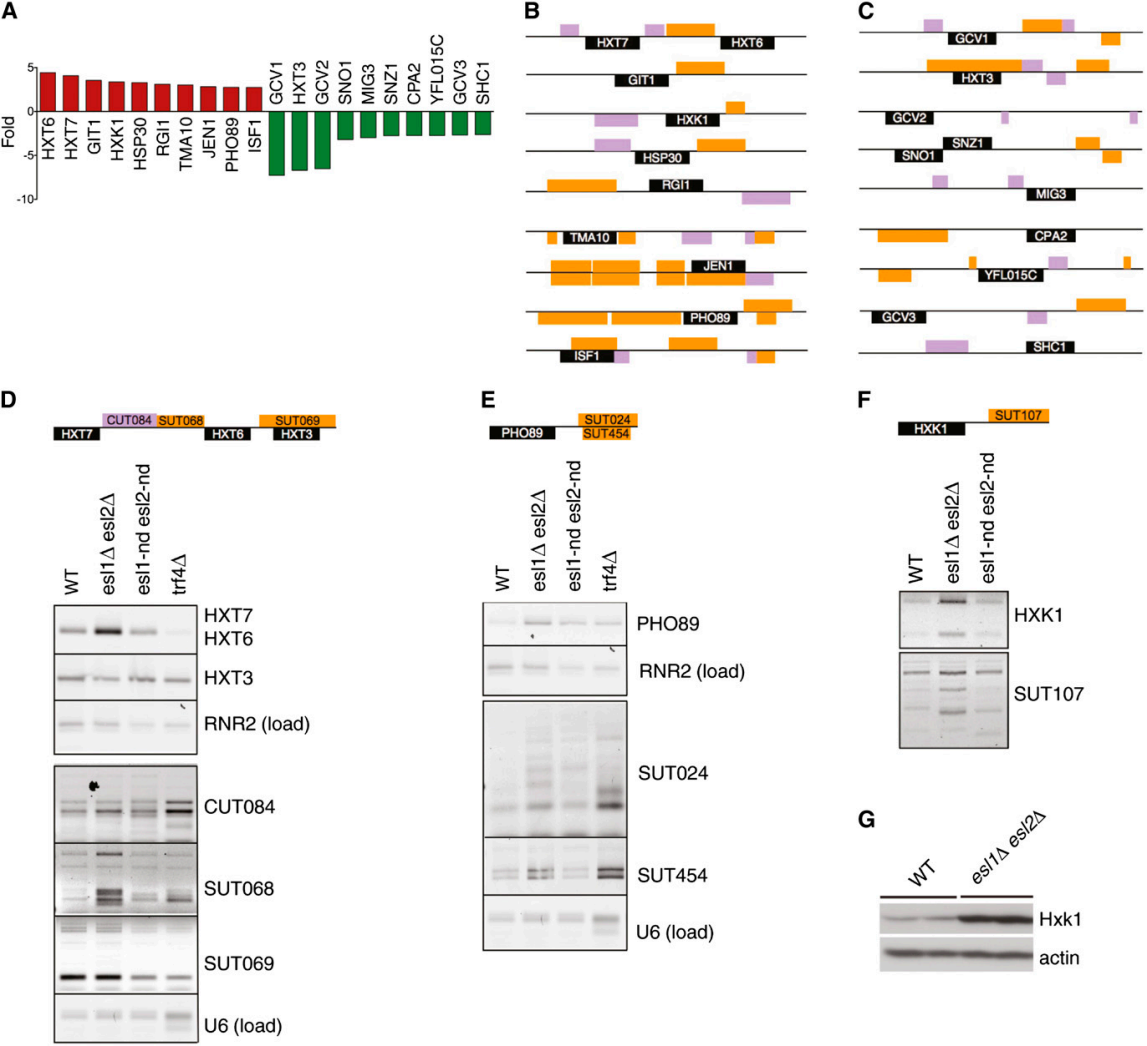
Gene expression changes in *esl1Δ esl2Δ* mutants. (A) Graph showing fold changes of the 10 most highly upregulated and the 10 most highly downregulated transcripts from the microarray. (B and C) Schematic illustration of genomic loci of the top 10 upregulated (B) and top 10 downregulated (C) genes from the microarray (black) with neighboring cryptic unstable transcripts (CUTs; purple) and stable unannotated transcripts (SUTs; orange). (D–F) Reverse-transcriptase polymerase chain reaction (PCR) analysis of the expression levels of various transcripts of the indicated genotypes. Schematic diagrams of the genomic loci of analyzed transcripts are shown above each gel. (G) Western analysis of Hxk1 levels of two independent clones of the indicated genotypes. WT, wild-type.

Based on the genetic interaction with *trf4*Δ and the presumed PIN-mediated RNase function of Esl1/2, we performed a genome-wide RNA microarray analysis of *esl1Δ esl2Δ* double mutants compared with the wild-type to identify potential Esl1 and Esl2 targets. In total, the expression levels of 53 genes were altered by at least two-fold in *esl1Δ esl2Δ* cells, with 30 genes that were upregulated and 23 genes that were downregulated (Figure 5). The most highly enriched gene ontology terms associated with the deregulated genes include glycine metabolic process and carbohydrate transport (G0:0006544 and GO:0008643, each with adjusted p < 0.001). The 10 most highly upregulated or downregulated genes are indicated in Figure 6A, and their genomic contexts are shown in Figure 6, B and C. Upregulated expression was confirmed by semiquantitative reverse-transcription PCR analyses for the transcripts of *HXT6/HXT7* (which are too similar to be distinguishable by reverse-transcription PCR; Figure 6D), *PHO89* (Figure 6E), and *HXK1* (Figure 6F). In case of Hxk1, a similar upregulation of the protein was confirmed for two independent *esl1Δ esl2Δ* cultures compared with the wild-type by Western blot analysis (Figure 6G).

The most represented gene ontology terms among deregulated transcripts in *esl1Δ esl2Δ* mutants are in the classes of transport, carbohydrate metabolism, and one-carbon metabolism (Figure 5). Several hexose transporters involved in glucose uptake were deregulated in *esl1Δ esl2Δ* mutants. Strikingly, the high-affinity glucose transporters *HXT6* and *HXT7*, which are normally induced under low glucose conditions, were upregulated during growth in high-glucose medium, whereas the low-affinity transporter *HXT3*, which is normally expressed under high glucose conditions, was downregulated (Figure 5). Likewise, the hexokinase Hxk1 (Figures 5 and 6, F and G) and several other genes that are usually repressed in high glucose were derepressed in our mutants, including the *MAL* genes (*MAL12*, *MAL32*, and *MAL33*) that are involved in maltose transport and metabolism, the lactate transporter *JEN1*, and genes involved in glycogen metabolism (*GPH1* and *PGM2*). Similarly, even though the cells were grown in the presence of high levels of glycine, several transcripts of the one-carbon regulon—the glycine decarboxylase complex (*GCV1*, *GCV2*, and *GCV3*), the aminocarboxamide ribotide transformylase *ADE17*, and the cytoplasmic serine hydroxymethyltransferase *SHM2*—were downregulated, a phenomenon that is usually only observed on withdrawal of glycine from the environment (Subramanian *et al.* 2005). Similar aberrations were observed for genes involved in pyridoxine metabolism (*SNZ1* and *SNO1*), amino acid biosynthesis (*ARG3* and *CPA2*), and the ribosomal protein 18B (*RPL18B*), which previously have been reported to be coregulated with the genes involved in one-carbon metabolism (Gelling *et al.* 2004). Taken together, the data indicate that *esl1Δ esl2*Δ double mutants may have a defect in adapting the expression of hexose and one-carbon metabolism genes to environmentally appropriate requirements.

To determine if Esl1 and Esl2 directly regulate these transcripts via their PIN domains, we performed similar reverse-transcriptase PCR analyses of selected upregulated transcripts using the nuclease-dead mutant alleles. However, in the case of *HXT6/7* and *HXK1*, transcript levels were not altered in the nuclease-deficient alleles compared with the wild-type (Figure 6, D and F); in the case of *PHO89*, upregulation in *esl1-nd esl2-nd* mutants was attenuated compared with *esl1Δ esl2*Δ (Figure 6E). Thus, these data indicate that Esl1 and Esl2 may regulate the expression of the altered transcripts in a largely nuclease-independent manner.

### Nuclease-independent deregulation of noncoding transcripts in *esl1Δ esl2Δ* mutants

During the analysis of the 10 most highly upregulated or downregulated transcripts in *esl1Δ esl2*Δ mutants (Figure 6A), we noticed a striking association of these genes with neighboring noncoding so-called cryptic unstable transcripts (CUTs) and stable unannotated transcripts (SUTs) (Figure 6, B and C). Because some of these noncoding RNAs are normally degraded in a TRAMP complex–dependent manner (Xu *et al.* 2009), we wondered whether deregulation of SUTs and CUTs might explain the synthetic sickness of *esl1Δ esl2*Δ with *trf4*Δ (Figure 4A). Interestingly, reverse-transcriptase PCR analysis confirmed upregulation of most of the CUTs and SUTs near *HXT7/HXT6*, *PHO89*, and *HXK1* loci in *esl1Δ esl2Δ* cells (Figure 6, D–F). However, these changes in transcript levels were again independent of Esl1 and Esl2 nuclease domain integrity (Figure 6, D–F). For comparison, loss of *TRF4* seemed to have an overall similar effect as *esl1Δ esl2Δ* on the expression of the adjacent CUTs and SUTs but did not affect the expression of the Esl1-regulated and Esl2-regulated coding genes (Figure 6, D and E). Thus, the data suggest that deregulation of adjacent CUTs and SUTs is not directly linked to the expression levels of Esl1-regulated and Esl2-regulated coding genes.

## Discussion

Here, we have shown that Esl1 and Esl2 share extensive sequence similarity and a similar domain topology with hEST1A/B (SMG5/6). Moreover, given that the “original” yeast hEST1A/B-homolog Est1 lacks the defining PIN domain (Figure 1), Esl1 and Esl2—at least from a structural perspective—may be considered to be the “real” orthologs of hEST1A/B. However, despite these structural similarities, several independent lines of experimental evidence indicated that Esl1 and Esl2 are seemingly neither involved in telomere length and structural maintenance mechanisms nor involved in NMD-related functions in yeast. Instead, the findings that loss of Esl1 and of Esl2 lead to synthetic sickness with two different components of the Rim101 pH-sensing pathway (Figure 4B) and to upregulation or downregulation of glucose and amino acid metabolic genes in the opposite direction as physiological requirements (Table 1 and Figure 5) indicate that these two PIN domain proteins might contribute to environment-sensing adaptive transcriptional response mechanisms.

Dfg16 and Rim8 regulate the proteolytic cleavage of the Rim101 transcription factor under alkaline conditions to facilitate its nuclear translocation and activation of pH-responsive genes (Lamb and Mitchell 2003; Lamb *et al.* 2001). While our work was in progress, the *RIM9*, *RIM13*, and *RIM20* genes involved in the Rim101 pH-responsive pathway were identified as genetic interactors of *ESL2* in another genome-wide synthetic genetic interaction screen (Costanzo *et al.* 2010), further supporting that Esl1 and Esl2 may function in a pathway parallel to Rim101. Interestingly, the set of genes regulated in response to alkaline conditions overlaps considerably with the response to low glucose conditions (Ruiz *et al.* 2008; Serrano *et al.* 2006; Viladevall *et al.* 2004), particularly with regard to glucose-repressed genes involved in glucose utilization and carbohydrate metabolism. Consistent with this, a high proportion of deregulated transcripts in *esl1Δ esl2Δ* mutants is involved in hexose transport, lactose transport, and carbohydrate metabolism (*e.g.*, *HXT13*, *HXT3*, *HXT6*, *HXT7*, *JEN1*, *HXK1*, *PGM2*, *MAL12*, *MAL32*, and *MAL33*; Table 1), and should have been repressed under the high glucose conditions during the experiment. At the same time, another set of genes involved in one-carbon metabolism (*GCV1*, *GCV2*, *GCV3*, *SHM2*, and *ADE17*) as well as some coregulated transcripts such as genes involved in pyridoxine metabolism (*SNZ1* and *SNO1*) and amino acid biosynthesis (*ARG3* and *CPA2*), which are normally downregulated in response to glycine withdrawal (Gelling *et al.* 2004; Subramanian *et al.* 2005), were found to be downregulated under growth conditions with sufficient glycine in the medium (Table 1). Altogether, these findings indicate that *esl1Δ esl2Δ* mutants are defective in sensing or regulating the adaptive response to environmental growth conditions.

*Saccharomyces cerevisiae* contains seven PIN domain–containing proteins: Nob1 and Utp24, which are involved in ribosome biogenesis by assisting in endonucleolytic cleavage of rRNA precursors (Bleichert *et al.* 2006; Fatica *et al.* 2003; Fatica *et al.* 2004); Nmd4, which was isolated in a two-hybrid screen for Upf1-interacting proteins but whose role in NMD remains to be determined (He and Jacobson 1995); Swt1, which has *bona fide* endoribonuclease activity and contributes to mRNA quality-control at the nuclear pore complex (Rother *et al.* 2006; Schaeffer *et al.* 2009; Skruzny *et al.* 2009); Rrp44, which forms part of the core of the nuclear and cytoplasmic RNA processing exosome complex and exhibits exoribonuclease activity (Dziembowski *et al.* 2007; Liu *et al.* 2006) as well as endoribonuclease activity *in vitro* (Schaeffer *et al.* 2009); and the previously uncharacterized related proteins Esl1 and Esl2. Although the conceptual connection to environment-sensing adaptive response pathways may explain the synthetic sickness of *esl1Δ esl2Δ* mutants with *dfg16Δ* and *rim8Δ* (Figure 4B), the reason for their synthetic sickness with *trf4Δ* is less clear (Figure 4A). A surprising finding was that the most highly deregulated transcripts in *esl1Δ esl2Δ* mutants were associated with similarly deregulated noncoding RNAs (Figure 6). Because Trf4 is a component of the TRAMP complex involved in the degradation of at least some of these RNAs, a possible explanation could be that the combination of increased expression and reduced degradation of CUTs and SUTs leads to reduced genetic fitness of *esl1Δ esl2Δ trf4*Δ triple mutants. Similarly, the reasons for increased mitochondrial genome instability and altered drug sensitivities in *esl1* and *esl2* mutants (Figure 2) also remain to be determined. However, mitochondrial DNA integrity is very sensitive to fluctuations in cellular dNTP levels (Zhao *et al.* 1998), and it is conceivable that dNTP homeostasis would be affected by impaired hexose metabolism in *esl1Δ esl2*Δ mutants.

Budding yeast are able to proliferate and survive in diverse and often rapidly changing environments—for example, during the fermentation process with quickly changing glucose, ethanol, and acidity levels—but the adaptive response mechanisms remain poorly understood. Our study indicates that the previously uncharacterized Esl1 and Esl2 proteins may be involved in this process and provide a basis for future investigations into the detailed mechanisms by which they exert this function as well as investigations into the roles of their nuclease domains in these and other processes.
